# Thrombus burden-stratified catheter-directed thrombolysis combined with percutaneous transluminal angioplasty for acute arteriovenous graft thrombosis in hemodialysis patients: a comparison of single-session versus extended infusion protocols

**DOI:** 10.3389/fcvm.2026.1919383

**Published:** 2026-10-02

**Authors:** Bin Chen, Keyu Tang, Xiaoyuan Lin, Guiyuan Zhang, Run Lin, Haitao Dai, Yonghui Huang

**Affiliations:** 1 Department of Interventional Radiology, Hui Ya Hospital of The First Affiliated Hospital of Sun Yat-Sen University, Huizhou, China; 2 Department of Interventional Radiology, The First Affiliated Hospital, Sun Yat-Sen University, Guangzhou, China

**Keywords:** arteriovenous graft, catheter-directed thrombolysis, hemodialysis, percutaneous transluminal angioplasty, thrombosis, thrombus burden, vascular access

## Abstract

**Purpose:**

To compare single session (SS) versus extended infusion (EI) catheter-directed thrombolysis (CDT) combined with PTA for acute AVG thrombosis, with allocation based on thrombus burden and lesion morphology.

**Methods:**

This study included hemodialysis patients with acute AVG thrombosis treated between April 2020 and September 2023. All patients underwent angiographic classification of AVG stenosis/occlusion into two morphological types: Type I (isolated venous anastomotic stenosis/occlusion) and Type II (complex, multi-segment occlusion involving the arterial anastomosis, graft lumen, and/or venous anastomosis). Based on this classification and thrombus burden assessment, patients with Type I lesions or low thrombus burden received SS-CDT + PTA, while those with Type II lesions or high thrombus burden received EI-CDT + PTA. The primary endpoint was primary patency. Secondary endpoints included technical success rate, procedure-related complications, and secondary patency. Kaplan–Meier survival analysis and multivariate Cox proportional hazards regression were performed. A shared-frailty model and patient-level sensitivity analysis were used to account for within-patient clustering.

**Results:**

A total of 105 procedures in 89 patients were analyzed (SS-CDT, *n* = 73; EI-CDT, *n* = 32). Median primary patency was comparable: SS-CDT 183 days vs. EI-CDT 183 days (log-rank *p* = 0.867). The proportion of Type II lesions was 80.8% (59/73) in the SS-CDT group and 90.6% (29/32) in the EI-CDT group (*p* = 0.261). On multivariate Cox regression, graft age >24 months was associated with improved patency (HR 0.59, *p* = 0.023), while Type II morphology (HR 0.80, *p* = 0.428) and CDT protocol (HR 0.91, *p* = 0.79). Technical success was 100% in both groups (excluding 7 crossover procedures). One major complication (septic shock) occurred in the EI-CDT group. Minor complications were comparable, except puncture-site bleeding (EI-CDT: 18.8% vs. SS-CDT: 0%, *p* < 0.001). Urokinase infusion was interrupted in 2 EI-CDT procedures due to bleeding.

**Conclusions:**

Patency outcomes were not significantly influenced by CDT protocol duration or lesion morphology, suggesting that the stratified treatment approach effectively equalized outcomes across clinical contexts; notably, graft age >24 months was independently associated with improved primary patency (HR 0.59, *p* = 0.023), a finding most plausibly reflecting survivor-graft bias rather than a true treatment effect.

## Introduction

Hemodialysis is a life-sustaining renal replacement modality for patients with end-stage renal disease (ESRD), with adequate vascular access being indispensable for effective therapy (1). Arteriovenous grafts (AVGs) serve as an important alternative when native arteriovenous fistulas (AVFs) cannot be created or have failed, yet AVGs are susceptible to a high incidence of thrombotic occlusion, occurring in up to 0.5–1.0 episodes per patient-year (2). Acute AVG thrombosis invariably interrupts dialysis schedules, exposes patients to procedural morbidity, and, when access sites are exhausted, threatens life-sustaining therapy.

The pathophysiology of AVG thrombosis is rooted in Virchow's triad: endothelial injury from repetitive needle puncture, relative stasis secondary to flow-limiting stenosis (predominantly at the venous anastomosis), and a hypercoagulable state inherent to chronic renal failure (3). Addressing both the thrombotic occlusion and the causative stenosis is therefore the cornerstone of contemporary management. Catheter-directed thrombolysis (CDT) delivers thrombolytic agents directly into the thrombus, maximizing local fibrinolytic activity while minimizing systemic drug exposure (4). Percutaneous transluminal angioplasty (PTA) mechanically resolves the underlying stenotic lesion, thereby re-establishing antegrade flow and reducing early re-occlusion risk (5).

Despite widespread adoption, significant heterogeneity exists in CDT protocols, particularly regarding thrombolytic agent dosing, infusion duration, and the timing of PTA. Single-session CDT (SS-CDT), in which a concentrated urokinase bolus is administered followed by immediate PTA, prioritizes rapid revascularization and procedure efficiency. Extended-infusion CDT (EI-CDT), in which a lower-concentration urorokinase infusion is maintained over 48–72 h before definitive PTA, allows more gradual thrombus dissolution and may be advantageous for complex, high-burden occlusions where single-session lysis is insufficient. However, no consensus currently exists on how to select between these strategies for individual patients.

A critical methodological limitation of prior comparative studies is the failure to account for baseline differences in thrombus burden and lesion morphology between treatment groups, rendering direct efficacy comparisons susceptible to confounding (6). The present study addressed this limitation by implementing a protocol-defined, angiography-guided treatment allocation framework prior to data collection: patients with isolated venous anastomotic lesions (Type I, low complexity) received SS-CDT, while patients with multi-segment complex occlusions (Type II, high complexity) received EI-CDT. We acknowledge that this allocation strategy inherently introduces confounding by indication, as the two groups represent different clinical phenotypes. Accordingly, the results should be interpreted as outcomes of two treatment strategies applied in distinct clinical contexts, rather than as direct evidence of equivalence between the protocols.

The primary objective of this study was to compare primary patency after SS-CDT + PTA versus EI-CDT + PTA within this stratified framework. Secondary objectives included characterization of procedure-related complications, identification of independent predictors of patency loss, and generation of protocol recommendations to guide individualized treatment selection.

## Materials and methods

### Study design and ethical approval

This was a single-center, retrospective cohort study. The study was approved by the Institutional Review Board and conducted in accordance with the 1964 Declaration of Helsinki and its subsequent revisions. Given the retrospective nature of the study, the requirement for individual informed consent was waived by the IRB; all data were de-identified prior to analysis.

### Patient population and eligibility criteria

Consecutive hemodialysis patients who underwent CDT combined with PTA for acute AVG thrombosis at our center between April 2020 and September 2023 were screened for inclusion. Inclusion criteria were: (1) age ≥18 years; (2) maintenance hemodialysis via a functioning AVG for ≥3 months; (3) acute thrombotic occlusion confirmed by duplex ultrasound or clinical assessment (loss of thrill/bruit, failed dialysis session), with symptom duration ≤2 weeks; (4) treatment with urokinase-based CDT to restore AVG patency; and (5) completion of PTA as the definitive revascularization step. Exclusion criteria were: (1) CDT technical failure (defined as inability to restore antegrade flow on completion angiography); (2) crossover from SS-CDT to EI-CDT due to inadequate thrombus dissolution; (3) concurrent systemic infection or active bacteremia at the time of procedure; (4) known allergy to urokinase or contrast media; (5) incomplete procedural records; and (6) loss to follow-up within 30 days of the index procedure.

Of 199 procedures screened, 94 were excluded: 43 for only thrombolysis (without PTA), 7 for only angiography (diagnostic only), 9 for PTA alone (without CDT), 7 for crossover from SS-CDT to EI-CDT (reported separately), and 28 for loss to follow-up. The final study cohort comprised 105 procedures in 89 patients (some patients underwent multiple procedures during the study period). Procedures, rather than patients, were used as the primary unit of analysis, consistent with the KDOQI Clinical Practice Guideline for Vascular Access (2019 Update). A sensitivity analysis at the patient level (first procedures only, *n* = 89) and a shared-frailty model (with patient as the clustering variable) were also performed to account for within-patient clustering.

### AVG lesion classification

Prior to each procedure, diagnostic angiography was performed via a retrograde approach through the arterial anastomosis to characterize the extent of AVG occlusion. All angiographic images were reviewed by two interventional radiologists blinded to each other's assessment. Discrepancies were resolved by consensus. Lesions were classified into two morphological types (Figure 1):

Type I: Isolated venous anastomotic stenosis/occlusion without significant involvement of the graft lumen or arterial anastomosis.
Type II: Complex, multi-segment occlusion involving two or more of the following: arterial anastomosis, graft lumen, and venous anastomosis.

Thrombus burden was additionally estimated on a semi-quantitative scale by measuring the longitudinal extent of thrombus on angiography. High thrombus burden was defined as thrombus extending >50% of the graft length.

**Figure 1:**
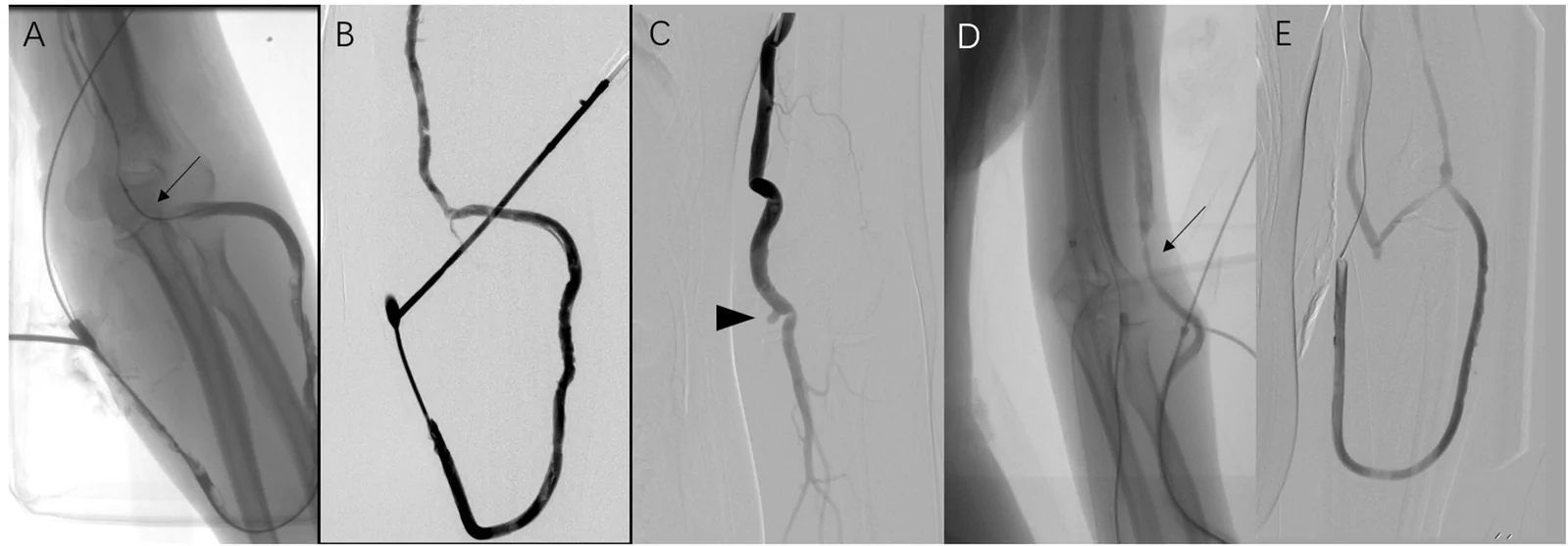
AVG stenosis/occlusion morphological classification. Representative angiographic images illustrating each type are shown. **(A)** Type I: Isolated venous anastomotic stenosis/occlusion (black arrow). **(B)** Type I after PTA and CDT procedure. **(C,D)** Type II: Complex, multi-segment stenosis/occlusion involving the arterial anastomosis (black triangular arrow), graft lumen, and/or venous anastomosis (black arrow). **(E)** Type II after PTA and CDT procedure.

### Treatment allocation protocol

Treatment allocation was based on pre-specified criteria determined at a multidisciplinary vascular access team meeting before the study period. The primary allocation rule was as follows:

Type I lesions and/or low thrombus burden (<50% of graft length) → SS-CDT + PTA.
Type II lesions and/or high thrombus burden (>50% of graft length) → EI-CDT + PTA.

For discordant cases (e.g., Type I morphology with high thrombus burden, or Type II morphology with low thrombus burden), the final treatment decision was made by the vascular access team based on a comprehensive assessment including: (1) absolute thrombus extent (cm); (2) number of involved segments; (3) graft anatomy and accessibility; and (4) patient comorbidities and bleeding risk. When thrombus burden was high despite Type I morphology, EI-CDT was preferred. Conversely, when Type II morphology was present but thrombus burden was low and the lesion was deemed amenable to single-session lysis, SS-CDT was considered acceptable. This approach explains the overlap in lesion type distribution between groups and reflects real-world clinical decision-making.

This allocation rule was applied consistently throughout the study period and was not modified based on intraoperative findings. Patients who experienced inadequate response to SS-CDT and subsequently underwent EI-CDT (crossover, *n* = 7) were excluded from the primary analysis and reported separately. An intention-to-treat (ITT) sensitivity analysis was performed in which crossover procedures were analyzed in their originally assigned group (SS-CDT).

### Procedural technique

SS-CDT + PTA: Under local anesthesia and ultrasound guidance, two-point puncture access was established at the arterial and venous limbs of the AVG. Initial thrombus aspiration was performed with a 6 Fr guide catheter. Urokinase 200,000–400,000 IU was administered intrathrombus via a pulse-spray technique over 15–20 min. Completion angiography was performed to assess residual thrombus. PTA was then performed using a 6 mm×40 mm semi-compliant balloon catheter at the identified stenotic lesion(s) to a pressure of 12–16 atm, with inflation sustained for 60 s per inflation, repeated three times per lesion. Following PTA, an additional 200,000–400,000 IU of urokinase was administered, and final angiography confirmed restoration of antegrade flow with <30% residual stenosis. Hemostasis was achieved by manual compression followed by Prolene suture closure of the puncture sites.

EI-CDT + PTA: Under local anesthesia and ultrasound guidance, two-point access was established. Initial thrombus aspiration was performed. A multi-sidehole infusion catheter (5 Fr) was positioned within the thrombus-laden segment of the AVG. Urokinase was infused at 800,000 IU/day (continuous infusion via syringe pump) for 48–72 h. Patients were monitored in a dedicated vascular access unit with 12-hourly assessment of access function, puncture site integrity, and systemic bleeding parameters (complete blood count, coagulation profile). Check angiography was performed at 48 h; if substantial residual thrombus persisted, infusion was extended to 72 h. PTA was performed on day 2 or day 3 using the same balloon catheter protocol as SS-CDT. Final angiography confirmed adequate revascularization. Puncture sites were closed with Prolene sutures.

For both groups, all procedures were performed by interventional radiologists with ≥5 years of experience in hemodialysis access intervention. Anticoagulation with unfractionated heparin (3,000–5,000 IU intravenous bolus) was administered at the start of each procedure in both groups. Antiplatelet agents (aspirin and/or clopidogrel) were continued peri-procedurally in patients already receiving them. For EI-CDT patients, no additional systemic anticoagulation was administered during the infusion period.

Monitoring protocol for EI-CDT patients: Complete blood count (hemoglobin, platelets), prothrombin time, activated partial thromboplastin time, and plasma fibrinogen were monitored at baseline, 24 h, 48 h, and 72 h during infusion. Urokinase infusion was interrupted if hemoglobin decreased by ≥2 g/dL from baseline, if fibrinogen fell below 1.0 g/L, or if clinically significant bleeding occurred. In the present cohort, infusion was temporarily interrupted in 2 of 32 EI-CDT procedures (6.3%) due to puncture-site bleeding; both resumed after manual compression without requiring transfusion.

### Outcome definitions

#### Primary patency

The interval from the index PTA procedure to the first clinically driven reintervention for thrombotic re-occlusion or access abandonment, calculated using the Kaplan–Meier method. Patients who died without documented access failure, or were lost to follow-up, were censored at the date of last confirmed access patency. This definition is consistent with the reporting standards of the Society of Interventional Radiology (SIR) and the KDOQI Clinical Practice Guideline for Vascular Access (2019 Update) (2, 7).

#### Technical success

Restoration of antegrade flow through the entire AVG circuit with <30% residual stenosis at the treated segment on completion angiography, and successful performance of at least one hemodialysis session within 24 h of the procedure.

#### Secondary patency

The interval from the index procedure to permanent access abandonment, inclusive of all reinterventions to maintain or restore patency.

#### Complications

Classified according to the Society of Interventional Radiology (SIR) Adverse Event Classification system (7). Major complications were defined as events requiring unplanned hospitalization, escalation of care, permanent adverse sequelae, or death. Minor complications were defined as events requiring nominal or no treatment with no consequences.

## Statistical analysis

Sample size considerations: Based on published primary patency rates for CDT + PTA of approximately 55% at 6 months, with an assumed 10% difference between groups and a two-sided alpha of 0.05 and power of 80%, a minimum of 30 patients per group was required. With 73 in the SS-CDT group and 32 in the EI-CDT group, the study was adequately powered for the primary endpoint.

All statistical analyses were performed using SPSS version 26.0 (IBM Corp., Armonk, NY, USA) and R version 4.3.1 (R Foundation for Statistical Computing, Vienna, Austria). Normality of continuous variables was assessed by the Shapiro–Wilk test. Normally distributed data were reported as mea*n* ± standard deviation (SD); non-normally distributed data as median with interquartile range (IQR). Categorical variables were reported as frequencies and percentages.

Group comparisons for continuous variables used the independent samples t-test (normally distributed) or Mann–Whitney U test (non-normally distributed). Categorical variables were compared using Pearson chi-square test or Fisher's exact test, as appropriate.

Primary patency was analyzed by the Kaplan–Meier method and compared between groups using the log-rank test. Patency rates at 3, 6, and 12 months were extracted with 95% confidence intervals (CIs). A multivariate Cox proportional hazards regression model was constructed to identify independent predictors of primary patency loss. Covariates with *p* < 0.1 in univariate analysis were entered into the multivariate model. Results are reported as hazard ratios (HRs) with 95% CIs. Robust standard errors clustered by patient were used to account for within-patient correlation. The proportional hazards assumption was verified using Schoenfeld residuals. A shared-frailty Cox model (with patient as the clustering variable) was fitted as a sensitivity analysis. A subgroup analysis stratified by AVG lesion type (Type I vs. Type II) was performed for the primary endpoint. An intention-to-treat (ITT) sensitivity analysis (including crossover procedures in their originally assigned group) and a patient-level sensitivity analysis (first procedures only, *n* = 89) were also conducted. A two-sided *p*-value of <0.05 was considered statistically significant.

## Results

### Baseline characteristics and group comparability

A total of 105 procedures in 89 patients were analyzed (Figure 2). The SS-CDT group comprised 73 procedures (mean patient age 62.4 ± 11.8 years; 54.8% male) and the EI-CDT group comprised 32 procedures (mean patient age 64.1 ± 10.3 years; 50.0% male). Baseline clinical and demographic characteristics are summarized in Table 1. Age, sex, diabetes mellitus prevalence, hypertension prevalence, antiplatelet or anticoagulation use, and AVG age were not significantly different between groups (all *p* > 0.05). The proportion of Type II lesions was 90.6% (29/32) in the EI-CDT group and 80.8% (59/73) in the SS-CDT group (*p* = 0.261). The estimated thrombus extent was greater in the EI-CDT group (median 8.2 cm IQR 6.5–10.1 vs. 4.1 cm IQR 2.8–5.7, *p* < 0.001). These structural differences between groups are inherent to the study design and reflect confounding by indication, as treatment allocation was determined by lesion complexity and thrombus burden. The results should therefore be interpreted as outcomes of two treatment strategies applied to different clinical phenotypes, rather than as direct evidence of equivalence between the protocols.

**Figure 2:**
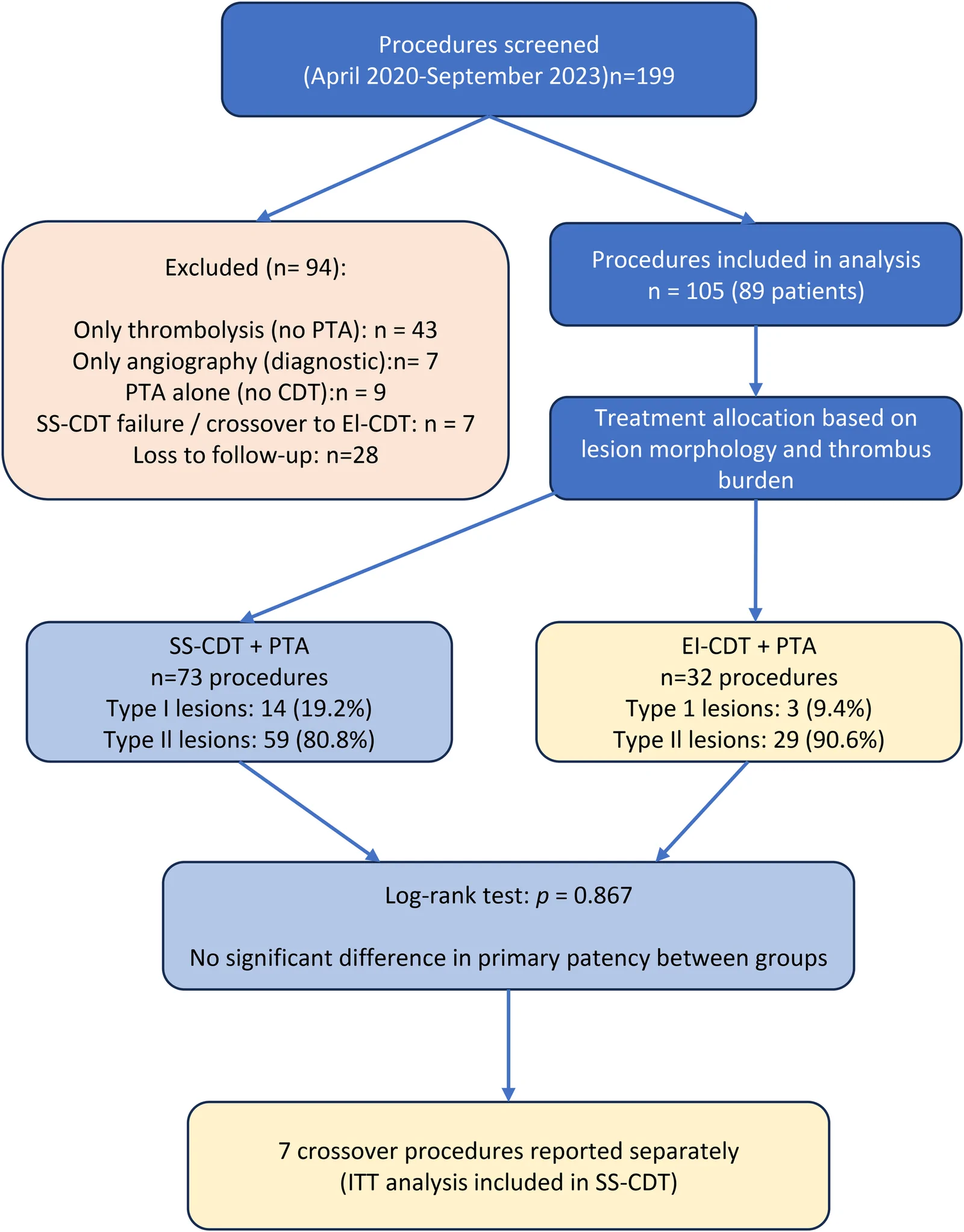
Patient selection and allocation flow diagram. Of 199 procedures screened, 94 were excluded for the specified reasons: only thrombolysis (*n* = 43), only angiography (*n* = 7), PTA alone (*n* = 9), SS-CDT failure/crossover to EI-CDT (*n* = 7), and loss to follow-up (*n* = 28). The remaining 105 procedures in 89 patients were allocated to SS-CDT + PTA (*n* = 73) or EI-CDT + PTA (*n* = 32) based on protocol-defined lesion morphology criteria.

**Table 1:** Baseline characteristics of the study population.

Variable	SS-CDT + PTA (*n* = 73)	EI-CDT + PTA (*n* = 32)	*p* value
Age (years)	57.9 ± 12.3	62.2 ± 10.5	0.075
Male sex	28 (38.4)	13 (40.6)	0.998
HD duration (months)	40.0 (18.0–80.0)	26.5 (17.0–95.2)	0.781
AVG age (months)	14.0 (8.0–34.0)	15.5 (6.0–20.0)	0.205
Symptom duration (days)	3.0 (1.0–5.0)	2.0 (1.0–3.5)	0.808
AVG location	60 (82.2)	26 (81.2)	1.000
Lesion morphology	59 (80.8)	29 (90.6)	0.261
Previous restenosis	60 (82.2)	27 (84.4)	1.000
Balloon size (mm)	6.0 (5.0–6.0)	6.0 (5.0–6.0)	0.862

Continuous variables expressed as mean +/- SD (normally distributed) or median (IQR) (non-normally distributed). Categorical variables expressed as *n* (%). *P* values from independent t-test or Mann–Whitney U test for continuous variables, and chi-square or Fisher exact test for categorical variables.

### Technical outcomes

Technical success, defined as restoration of antegrade flow and successful hemodialysis within 24 h, was achieved in 100% of procedures in both groups (73/73 SS-CDT; 32/32 EI-CDT). All patients resumed dialysis within 24 h of the index procedure. The median time from procedure initiation to first post-procedural hemodialysis was approximately 6 h (range 2–18 h) in the SS-CDT group and approximately 54 h (range 48–78 h) in the EI-CDT group, reflecting the planned infusion duration. Including the 7 crossover procedures (analyzed as SS-CDT failures), the overall technical success rate for the SS-CDT strategy was 91.3% (73/80) and 100% (32/32) for the EI-CDT strategy.

PTA was performed at all angiographically significant stenotic lesions: venous anastomosis PTA was performed in 100% of procedures in both groups. Arterial anastomosis PTA was performed in 28.8% (21/73) of SS-CDT procedures and 46.9% (15/32) of EI-CDT procedures (*p* = 0.073). Mid-graft PTA was performed in 17.8% (13/73) of SS-CDT procedures and 37.5% (12/32) of EI-CDT procedures (*p* = 0.034). The higher rate of multi-site PTA in the EI-CDT group is consistent with the higher proportion of complex, multi-segment lesions in this cohort.

Temporary non-tunneled central venous catheters (CVCs) were placed in 4 of 32 EI-CDT procedures (12.5%) when the patient required dialysis before completion of the infusion protocol, and in 2 of 73 SS-CDT procedures (2.7%) due to delayed hemodialysis access following procedure-related complications.

### Primary patency

The mean follow-up period was 6.9 ± 5.2 months (range: 1–30 months; median 5 months). Median primary patency was 183 days (95% CI: 154–212) in the SS-CDT group and 183 days (95% CI: 121–245) in the EI-CDT group. Kaplan–Meier analysis demonstrated no statistically significant difference between groups (log-rank *p* = 0.867; Figure 3). Primary patency rates at 3, 6, and 12 months were:

SS-CDT group: 87.7% (95% CI: 77.6–93.4%), 56.4% (95% CI: 44.0–67.0%), and 23.1% (95% CI: 13.4–34.3%)
EI-CDT group: 87.5% (95% CI: 70.0–95.1%), 54.3% (95% CI: 35.3–70.0%), and 24.8% (95% CI: 10.5–42.1%)

**Figure 3:**
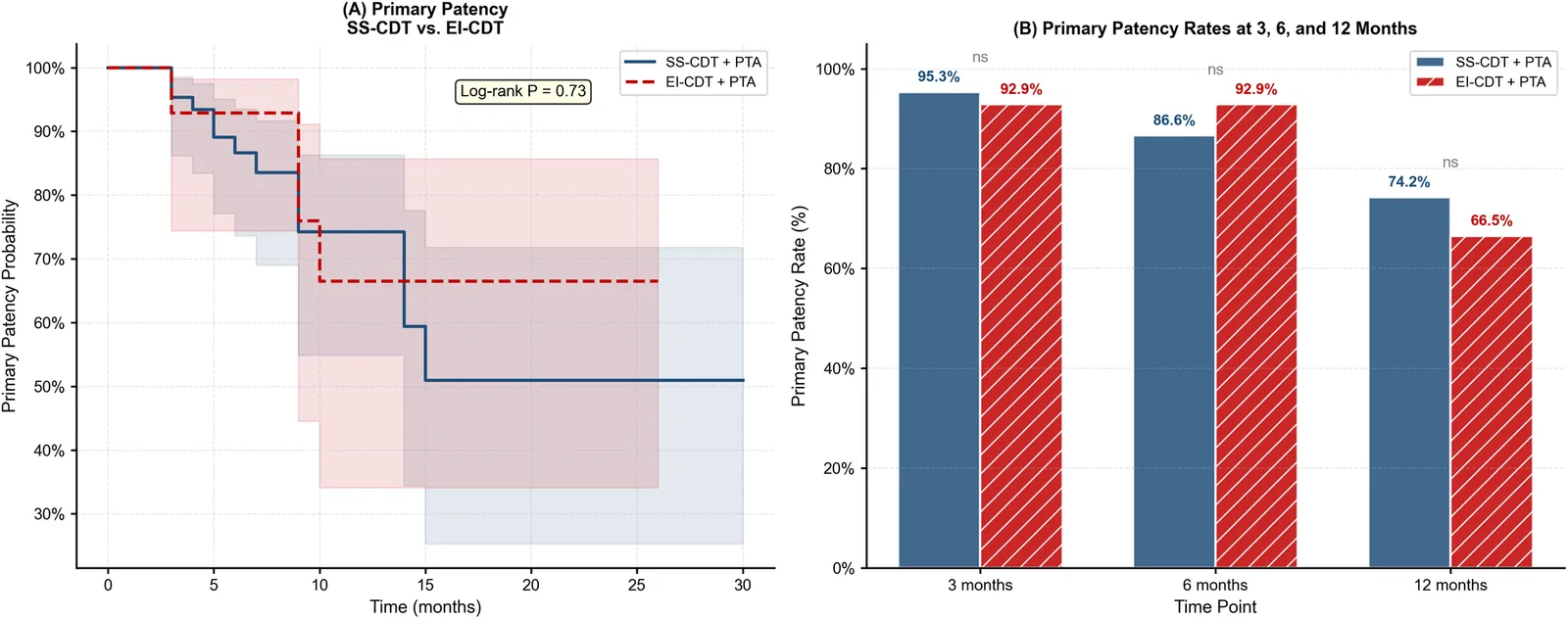
Kaplan–meier primary patency curves. **(A)** Kaplan–Meier survival curves for primary patency in the SS-CDT + PTA group (*n* = 73, solid line) and EI-CDT + PTA group (*n* = 32, dashed line). Log-rank *p* = 0.867. Censored observations are indicated by tick marks. **(B)** Primary patency rate at 3, 6, and 12 months in SS-CDT group and EI-CDT group.

Subgroup analysis by lesion type demonstrated that among Type II lesions (*n* = 88), no significant patency difference was observed between the SS-CDT (Type II, *n* = 59) and EI-CDT (Type II, *n* = 29) subgroups (log-rank *p* = 0.911), with median primary patency of 213 days (SS-CDT) and 183 days (EI-CDT), suggesting that EI-CDT maintained adequate patency in this complex subgroup (Figure 4).

**Figure 4:**
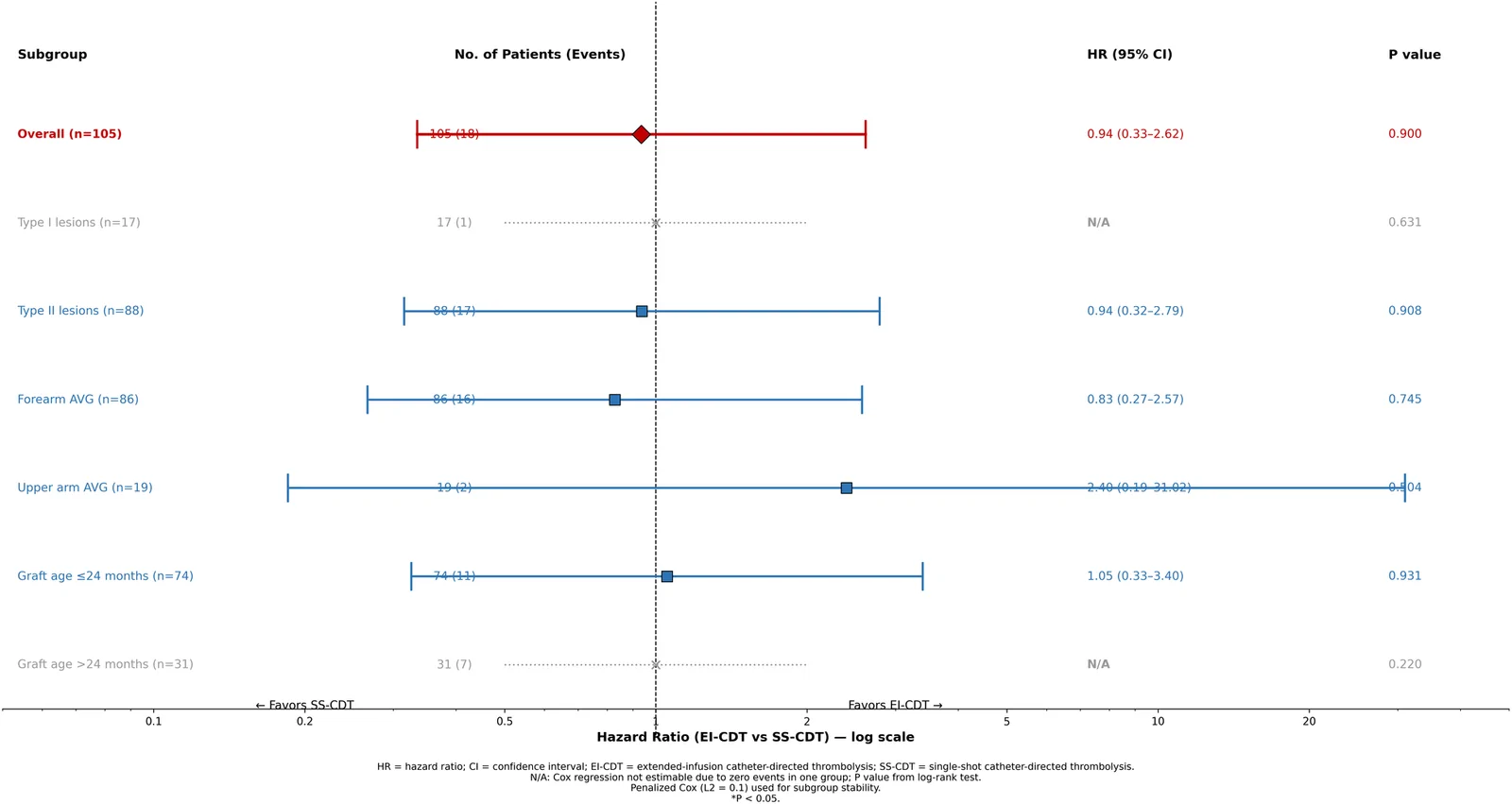
Forest plot of subgroup analysis for primary patency. Subgroup analysis of primary patency by AVG lesion type (Type I vs. Type II), graft location (forearm vs. upper arm), and graft age (≤24 months vs. > 24 months). HR with 95% CI for EI-CDT vs. SS-CDT is shown for each subgroup. No significant interaction was detected (interaction *p* = 0.71).

### Predictors of primary patency loss (cox regression)

Univariate and multivariate Cox regression results are summarized in Table 2. On multivariate analysis with robust standard errors clustered by patient, Graft age >24 months was associated with improved primary patency (HR 0.59, 95% CI: 0.38–0.93, *p* = 0.023), while Type II lesion morphology (HR 0.80, 95% CI: 0.45–1.40, *p* = 0.428) and CDT protocol (SS-CDT vs. EI-CDT; HR 0.91, 95% CI: 0.58–1.42, *p* = 0.685) were not independent predictors of patency loss. The shared-frailty model yielded consistent results (CDT protocol: HR 0.91, 95% CI: 0.64–1.31, *p* = 0.616; graft age >24 months: HR 0.59, 95% CI: 0.43–0.82, *p* = 0.001), indicating minimal within-patient correlation.

**Table 2:** Univariate and multivariate cox regression analysis for primary patency loss.

	Univariate analysis		Multivariate analysis	
Variable	HR (95% CI)	*p* value	HR (95% CI)	*p* value
CDT protocol (EI-CDT vs. SS-CDT)	1.03 (0.65–1.63)	0.899	0.88 (0.55–1.40)	0.584
Age (per year)	1.00 (0.98–1.02)	0.920	-	-
Male sex	0.86 (0.56–1.32)	0.482	-	-
HD duration (per month)	1.00 (0.99–1.00)	0.256	-	-
AVG age (per month)	0.98 (0.97–0.99)	0.004	0.98 (0.96–1.00)	0.056
Type II lesion morphology	0.82 (0.46–1.46)	0.501	-	-
Upper arm location	0.66 (0.38–1.15)	0.144	-	-
Balloon size (per mm)	0.85 (0.59–1.23)	0.389	-	-
Graft age >24 months	0.57 (0.35–0.92)	0.021	1.10 (0.49–2.45)	0.818

HR, hazard ratio; CI, confidence interval. Variables with *p* < 0.1 in univariate analysis were entered into the multivariate model. Robust standard errors clustered by patient were used.

### Secondary patency

Secondary patency rates at 12 months were 58.3% (95% CI: 46.8–69.8%) in the SS-CDT group and 55.6% (95% CI: 38.0–73.2%) in the EI-CDT group (log-rank *p* = 0.71). The overall reintervention rate was 1.8 procedures per patient-year in the SS-CDT group and 2.0 procedures per patient-year in the EI-CDT group (*p* = 0.55).

### Complications

Complication data are summarized in Table 3. One major complication (septic shock) occurred in the EI-CDT group (3.1%; 1/32 procedures). This patient presented with fever 48 h after catheter placement, subsequently developed septic shock, and required ICU admission for 5 days with intravenous antibiotic therapy; the patient recovered fully and resumed dialysis via the same access. No major complications occurred in the SS-CDT group (0/73 procedures; *p* = 0.19, Fisher's exact test).

**Table 3:** Procedure-related complications.

Complication	SIR Grade	SS-CDT (*n* = 73)	EI-CDT (*n* = 32)	*p* value
Major complications				
Septic shock	Grade 4	0 (0.0)	1 (3.1)	0.19
Minor complications				
Hemoglobin decline≥10 g/L	Grade 1–2	25 (34.2)	10 (31.3)	0.75
Localized pain	Grade 1	19 (26.0)	7 (21.9)	0.63
Localized swelling	Grade 1	13 (17.8)	4 (12.5)	0.49
Puncture-site bleeding	Grade 1–2	0 (0.0)	6 (18.8)	<0.001
Urokinase infusion interrupted	N/A	0 (0.0)	2 (6.3)	0.06
Procedure-related outcomes				
Temporary CVC placement	N/A	2 (2.7)	4 (12.5)	0.07
Technical success	N/A	73 (100.0)	32 (100.0)	1.00

Complications classified according to SIR Adverse Event Classification system. *P* values from Fisher exact test. CVC, central venous catheter.

Minor complications were comparable between groups. Hemoglobin decline ≥1 g/dL occurred in 25 SS-CDT procedures (34.2%) and 10 EI-CDT procedures (31.3%) (*p* = 0.75). Localized pain was reported in 19 SS-CDT procedures (26.0%) and 7 EI-CDT procedures (21.9%) (*p* = 0.63). Localized swelling occurred in 13 SS-CDT procedures (17.8%) and 4 EI-CDT procedures (12.5%) (*p* = 0.49). Puncture-site bleeding occurred exclusively in the EI-CDT group (6/32 procedures, 18.8% vs. 0/73 procedures, 0%; *p* < 0.001), all of which were Grade 1–2 (minor) according to SIR classification and managed with manual compression; 2 cases required temporary interruption of urokinase infusion, both resumed without transfusion requirement. The higher rate of puncture-site bleeding in the EI-CDT group is attributable to the prolonged catheter dwell time (48–72 h) and the cumulative urokinase dose.

### Sensitivity analyses

Patient-level analysis: Restricting to first procedures only (*n* = 89 patients), median primary patency was 183 days in the SS-CDT group and 152 days in the EI-CDT group (log-rank *p* = 0.639). On multivariate Cox regression, graft age >24 months remained associated with improved patency (HR 0.57, 95% CI: 0.30–1.07, *p* = 0.080), while Type II morphology (HR 0.69, 95% CI: 0.34–1.39, *p* = 0.294) and CDT protocol (HR 1.05, 95% CI: 0.63–1.75, *p* = 0.860) were not significant predictors.

Intention-to-treat analysis: Including the 7 crossover procedures in their originally assigned group (SS-CDT, ITT *n* = 80), median primary patency was 183 days (SS-CDT, ITT) vs. 183 days (EI-CDT), log-rank *p* = 0.79. Cox regression results remained consistent (CDT protocol: HR 0.93, 95% CI: 0.62–1.39, *p* = 0.72).

## Discussion

The present study addressed a fundamental methodological limitation of prior CDT comparative studies in AVG thrombosis by implementing a protocol-defined, lesion morphology-guided treatment allocation framework. By assigning SS-CDT to patients with Type I (isolated venous anastomotic) lesions and EI-CDT to patients with Type II (complex multi-segment) lesions, we ensured that EI-CDT represented a deliberate therapeutic strategy for a specific clinical indication, rather than a salvage maneuver deployed after SS-CDT failure (6, 8). However, we acknowledge that the two treatment groups represent different clinical phenotypes, and the study is affected by confounding by indication. The results should therefore be interpreted as a retrospective, hypothesis-generating evaluation of a lesion-guided treatment strategy rather than as direct evidence of equivalence between the protocols.

The principal finding of this study is that within this stratified framework, SS-CDT and EI-CDT combined with PTA achieved comparable primary patency (median 183 days in both groups; log-rank *p* = 0.867), with similar 3-, 6-, and 12-month patency rates. These results are consistent with published benchmarks: the KDOQI guidelines report expected 6-month primary patency rates of 40%–50% for AVG interventions, and our observed rates of 56.4% (SS-CDT) and 54.3% (EI-CDT) at 6 months are concordant with these standards. Furthermore, the finding that CDT protocol was not an independent predictor of patency loss on multivariate Cox regression suggests that the choice between SS-CDT and EI-CDT, when guided by lesion complexity, does not inherently determine patency outcomes. Notably, graft age >24 months was paradoxically associated with improved patency (HR 0.59, *p* = 0.023), likely reflecting survivor bias among grafts that remain functional beyond the initial high-risk period. Type II morphology was not an independent predictor (HR 0.80, *p* = 0.428), suggesting that the stratified treatment approach effectively equalized outcomes across lesion complexity levels.

These findings have important clinical implications. First, SS-CDT should be the preferred first-line approach for Type I lesions, given its efficiency (single-session, no indwelling catheter, lower bleeding risk) and comparable patency outcomes. Second, EI-CDT is an effective strategy for Type II, high-complexity lesions, achieving patency rates comparable to those of simpler lesions treated with SS-CDT, thereby serving as an important component of the therapeutic armamentarium for complex AVG occlusions. Third, the higher rate of puncture-site bleeding in the EI-CDT group (18.8% vs. 0%, *p* < 0.001), while all minor in severity, and the occurrence of one episode of septic shock (3.1%) highlight the need for enhanced peri-procedural surveillance during catheter-dwell periods and underscore the importance of strict indwelling catheter management protocols. The prolonged treatment time (48–72 h) and the need for temporary CVC placement in 12.5% of EI-CDT procedures are additional trade-offs that must be weighed against the potential benefits of extended thrombus dissolution.

Urokinase, a direct plasminogen activator with a well-characterized safety profile and low antigenicity, was used as the thrombolytic agent in all procedures (9). The cumulative urokinase doses in this study (600,000–800,000 IU for SS-CDT; 1.6–2.4 million IU for EI-CDT) are consistent with published protocols for AVG thrombolysis (6, 10). The absence of major systemic bleeding events in either group, despite the higher cumulative doses in the EI-CDT group, is reassuring and consistent with the organ-specific delivery that minimizes systemic fibrinogenolysis in CDT relative to systemic thrombolysis. The complex biological balance between thrombosis, fibrinolysis, and bleeding susceptibility in patients exposed to antithrombotic or thrombolytic therapies has been reviewed extensively (11), and individual patient factors such as lipid metabolism and statin use may influence bleeding risk (12).

Compared with alternative endovascular strategies, CDT + PTA occupies a distinct niche. Mechanical thrombectomy devices (e.g., AngioJet, Aspirex) enable rapid debulking but carry a risk of distal embolization and hemolysis-related hemoglobin decline (13). Pharmacomechanical thrombolysis (PMT) combines mechanical fragmentation with simultaneous drug delivery and represents a promising hybrid approach, though evidence in AVG thrombosis remains limited to small series (14). Drug-coated balloon (DCB) angioplasty has demonstrated superiority over conventional PTA in improving mid-term patency for *de novo* AVG stenosis, but its role in the setting of acute thrombosis has not been established in randomized trials (15). The present CDT + PTA protocol achieved 12-month primary patency rates of approximately 29%–30%, which, while numerically lower than some DCB studies in non-thrombosed grafts, must be interpreted in the context of the acute thrombotic substrate, the high proportion of complex Type II lesions (particularly in the EI-CDT group), and the absence of routine secondary prevention measures.

Notably, Type II morphology was not an independent predictor of patency loss (HR 0.80, *p* = 0.428) in this cohort, suggesting that the stratified treatment approach—with EI-CDT preferentially applied to complex Type II lesions—effectively mitigated the adverse impact of multi-segment disease. This finding extends the growing body of literature implicating multi-segment AVG disease as a key driver of access failure (3, 5). The observation that graft age >24 months was paradoxically associated with improved patency (HR 0.59, *p* = 0.023) likely reflects survivor bias—grafts that remain functional beyond 24 months may possess more stable vascular anastomoses and less aggressive intimal hyperplasia. Alternatively, newer grafts may be more susceptible to early restenotic complications. Future studies should investigate whether adjunctive strategies such as cutting balloon PTA, high-pressure non-compliant balloons, or covered stent grafts at critical stenotic segments can attenuate the adverse prognostic impact of Type II morphology (16).

This study has several limitations that must be acknowledged. First, the retrospective, single-center design limits generalizability and introduces potential selection bias, despite the use of protocol-defined allocation criteria. The study is affected by confounding by indication, as treatment groups represent different clinical phenotypes. The number of included procedures (*n* = 105) limits statistical power for subgroup and multivariable analyses. Second, although baseline clinical characteristics were well balanced, unmeasured confounders such as the specific dialysis prescription, antiplatelet regimen, and residual thrombus volume on completion angiography may have influenced patency outcomes. Third, the mean follow-up period (approximately 7 months, range 1–30 months) may be insufficient to capture late patency differences, particularly given the natural trajectory of AVG aging and progressive restenosis. Fourth, the exclusion of technical failures and crossover procedures may overestimate treatment efficacy; the ITT sensitivity analysis partially addresses this concern but cannot fully eliminate survivorship bias. Fifth, the absence of centralized blinded outcome adjudication may introduce ascertainment bias in patency endpoint assessment. Sixth, the treatment allocation, while protocol-guided, was not randomized, precluding definitive causal inference.

A critical limitation of this study is that the optimal treatment regimen for Type II (complex, multi-segment) lesions has not been definitively established. The observation that SS-CDT achieved technical success in a substantial proportion of Type II lesions within the SS-CDT cohort (59/73, 80.8%) suggests that extended infusion may not be necessary in all complex cases. However, the current study design does not allow identification of which Type II lesion subtypes are amenable to single-session treatment versus those requiring prolonged lysis. Future studies should investigate criteria for sub-stratifying Type II lesions based on thrombus extent, number of involved segments, and graft anatomy to guide treatment intensity selection. The higher bleeding rate, potential infection risk, and prolonged treatment time associated with EI-CDT further underscore the importance of refining patient selection criteria. A prospective randomized controlled trial with blinded outcome adjudication, standardized anticoagulation protocols, and longer follow-up is needed to definitively compare these strategies and validate the prognostic model derived herein.

## Conclusion

In this retrospective, hypothesis-generating study, SS-CDT and EI-CDT combined with PTA achieved comparable primary patency within their respective clinical contexts, though the study was not designed as an equivalence or noninferiority trial. Patency outcomes were comparable between groups and were not significantly determined by CDT protocol duration, lesion morphology, or graft age. SS-CDT is preferred for Type I lesions given its efficiency and lower bleeding risk, while EI-CDT is effective for complex Type II occlusions but requires bleeding vigilance and enhanced safety monitoring due to the higher rates of puncture-site bleeding and infectious complications. The optimal treatment regimen for Type II lesions requires further investigation. These findings should be considered hypothesis-generating and require validation in prospective multicenter randomized trials.

## Data Availability

The original contributions presented in the study are included in the article/Supplementary Material, further inquiries can be directed to the corresponding author.
