# Supplementary material for: Thrombus burden-stratified catheter-directed thrombolysis combined with percutaneous transluminal angioplasty for acute arteriovenous graft thrombosis in hemodialysis patients: a comparison of single-session versus extended infusion protocols
Source: Front Cardiovasc Med. 2026 Oct 2;13:1919383. doi: 10.3389/fcvm.2026.1919383 (PMC13672488; doi:10.3389/fcvm.2026.1919383)
Supplement: Supplementary file 1 [file Table1.docx]

Supplementary Table S1. Subgroup Analysis of Primary Patency

| **Subgroup** | **SS-CDT n (events)** | **EI-CDT n (events)** | **HR (95% CI)** | **P value** | **Method** |
| --- | --- | --- | --- | --- | --- |
| Overall | 73 (60) | 32 (27) | 1.03 (0.67-1.58) | 0.905 | Cox regression |
| Type I lesions | 14 (13) | 3 (3) | 0.71 (0.21-2.39) | 0.584 | Cox regression |
| Type II lesions | 59 (47) | 29 (24) | 1.02 (0.64-1.64) | 0.921 | Cox regression |
| Forearm AVG | 60 (48) | 26 (22) | 0.88 (0.54-1.42) | 0.593 | Cox regression |
| Upper arm AVG | 13 (12) | 6 (5) | 1.87 (0.61-5.74) | 0.277 | Cox regression |
| Graft age≥24 months | 47 (41) | 27 (22) | 0.88 (0.54-1.45) | 0.621 | Cox regression |
| Graft age > 24 months | 26 (19) | 5 (5) | 0.94 (0.33-2.63) | 0.902 | Cox regression |

*Note: HR for EI-CDT vs SS-CDT within each subgroup. "N/A" indicates Cox regression could not be performed due to zero events in one group; log-rank p value is reported instead.*

Supplementary Table S2. Sensitivity Analyses

| **Analysis** | **SS-CDT** | **EI-CDT** | **P value** |
| --- | --- | --- | --- |
| Primary analysis (all procedures) | n=73, median=183d | n=32, median=183d | 0.867 |
| Patient-level (first procedures only) | n=50, median=183d | n=24, median=152d | 0.696 |
| ITT analysis (crossovers in SS-CDT) | n=80, median=183d* | n=32, median=183d | ~0.64 |

*Note: *ITT analysis includes 7 crossover procedures (SS-CDT failure -> EI-CDT) in their originally assigned group (SS-CDT). P values from log-rank test.*
